# Nontoxic Goiter (NTG) and Radioiodine: What Do Patients Think About It? Quality of Life in Patients with NTG Before and After 131-I Therapy

**DOI:** 10.3389/fendo.2018.00114

**Published:** 2018-04-16

**Authors:** Sonia Kaniuka-Jakubowska, Anna Lewczuk, Mikołaj Majkowicz, Maciej Piskunowicz, Krystyna Mizan-Gross, Adam Zapaśnik, Mariusz Kaszubowski, Piotr Lass, Krzysztof Sworczak

**Affiliations:** ^1^Department of Endocrinology and Internal Medicine, Medical University of Gdansk, Gdansk, Poland; ^2^Department for Quality of Life Research, Medical University of Gdansk, Gdansk, Poland; ^3^Department of Public Health, Pomeranian University in Slupsk, Slupsk, Poland; ^4^Department of Radiology, Medical University of Gdansk, Gdansk, Poland; ^5^Department of Nuclear Medicine, Medical University of Gdansk, Gdansk, Poland; ^6^Department of Economic Sciences, Faculty of Management and Economics, Gdansk University of Technology, Gdansk, Poland; ^7^Institute of Experimental Physics, University of Gdansk, Gdansk, Poland

**Keywords:** nontoxic goiter, radioiodine, quality of life, 36-item Short Form, life quality

## Abstract

**Objective:**

Despite numerous publications regarding nontoxic goiter (NTG) treatment and an increasing interest in patients’ quality of life, few studies present the outcome of 131-I treatment from the patients’ perspective. Our study’s main aim was to verify whether there is any improvement in life quality following 131-I treatment.

**Materials and methods:**

Thirty-five patients with NTG qualified to participate in the study. All patients completed a Thyroid-Related Health-Related Quality of Life (Thy-R-HRQoL) questionnaire created by us and the Medical Outcomes Study 36-item Short Form (SF-36), right before and 1 year after 131-I.

**Results:**

We observed an improvement in six out of eight SF-36 and three out of seven Thy-R-HRQoL domains. In comparison with the control group, we observed worse results in two out of eight, prior to treatment, and one out of eight SF-36 afterward, as well as in all Thy-R-HRQoL domains. We did not find any correlation between improvement of Thy-R-HRQoL and SF-36 and goiter size reduction, except for Bodily Pain. There was also no correlation between improvement of SF-36 and Thy-R-HRQoL domains, and goiter size before treatment. The older the patient, the less noticeable improvement was observed in Physical and Social Functioning, and Vitality in SF-36, but age had no influence on the assessment by Thy-R-HRQoL.

**Conclusion:**

Radioiodine treatment improves life quality in patients with NTG. Use of the Health-Related Quality of Life questionnaire should be taken into consideration when evaluating life quality of patients with NTG. Relentless pursuit of maximal goiter size reduction in 131-I treatment is worth consideration. In our study, life quality improvement did not depend directly on the goiter size reduction. Life quality improvement after 131-I might not depend on initial goiter size, and for certain domains of SF-36 might be less clearly expressed in older patients.

## Introduction

Radioiodine (131-I) administration is an ideal alternative, especially in patients disqualified from surgical treatment or unwilling to undergo surgery. Good arguments for 131-I treatment are the short hospitalization period (or even no hospitalization in case of fractionated dosage), as well as lack of convalescence period and lack of potential perioperative complications. The vast majority of medical literature regarding nontoxic goiter (NTG) focuses on treatment methods and their efficacy (1, 2). The main aim of 131-I treatment is maximal reduction of thyroid volume (1, 2). It is known that 131-I administration reduces goiter size an average of 40–60% (1, 3). Recombinant human thyrotropin was recently used to enhance therapeutic results (4). It is worth posing the question of whether greater goiter size reduction truly leads to greater patient satisfaction. Consequently, is relentless pursuit of maximal thyroid volume reduction what patients expect from treatment? Last but not least, can patients with below median thyroid volume reduction find treatment results satisfactory and experience an improvement of their well-being as well? We attempt to answer these questions based on collected data.

### Aims

The aim of our study was to assess quality of life (QoL) of patients with NTG right before and 1 year after radioiodine treatment. Additionally, we attempted to verify whether there was any correlation between QoL improvement, the obtained goiter size reduction, goiter size before treatment, and patient’s age.

## Materials and Methods

Thirty-five patients with large NTG (34 women and 1 man), qualified for the study and received radioiodine to reduce goiter size. The Department of Endocrinology and Internal Medicine at the Medical University of Gdansk (MUG) was the head office of the study which was conducted between 2009 and 2012. The radioiodine treatment was administered at the Department of Nuclear Medicine of the same University. The scheme of the study and treatment gained consent from the MUG Independent Bioethics Commission.

Earlier, we published detailed characteristics of the group and the results of 131-I treatment in terms of goiter reduction and related complications (3).

Patient mean age was 65.63 (median: 66, range: 49.00–84.00). Patients received fractionated radioiodine with a single administered activity of 800 MBq (21.6 mCi). The total activity was calculated on the basis of iodine uptake and goiter size, according to a previously published formula (3). US scans, as a method comparable to CT scans, were used as a reference examination to assess goiter size and efficacy of treatment (5). Goiter size reduction was defined as a percentage, taking into account residual goiter volume 12 months after the treatment and the goiter’s initial size. Goiter size was estimated using the ellipsoid formula (5).

The control group consisted of 37 volunteers (acquaintances and family members of medical professionals employed by the Department). Mean age in this group was 68.35 (median 66; range 55.00–90.00).

All patients completed the Thyroid-Related and Generic Health-Related Quality of Life (HRQoL) survey right before and 1 year after treatment. Data collection was completed by handing out traditional paper questionnaires, asking the patients to fill them at home and return them to the researcher.

Generic HRQoL was assessed with the Polish version of Medical Outcomes Study 36-item Short Form (SF-36). The SF-36 consists of 36 questions divided into 8 domains: Physical Functioning (PF), Role-Physical (RP), Role-Emotional (RE), Vitality (VT), Mental Health (MH), Social Functioning (SF), Bodily Pain (BP), and General Health (GH). Patients could score between 0 and 100 points for each domain. The higher the score, the more satisfaction expressed, and the lower the score, the more apparent disability. The change in SF-36 test (SF-36 difference) was defined as the result of subtraction of scores after and before treatment. A positive difference indicated an improvement of SF-36 scale results.

To assess Thyroid-Related Thyroid-Related Health-Related Quality of Life (Thy-R-HRQoL), we prepared our own survey. The survey contained 49 questions and was divided into 7 domains: three focusing on the impact of (1) disease, (2) neck shape and deformities, (3) dyspnea on the GH and social role functioning; three of the domains evaluated the intensification of subjective symptoms: (4) breathing impairment, (5) sensation of a foreign body in the throat, (6) difficulty swallowing and hoarseness, and finally, (7) impact of the disease on the course of comorbidities. Each question could be answered with one of the following replies: definitely no, no, rather no, rather yes, yes, or definitely yes, and each answer could score between 1 and 6 points. Questions investigated whether different aspects of the disease influenced separate domains of the questionnaire. The higher the results of the test, the greater the impact of the disease/goiter on patient’s well-being. In this paper, each change in Thy-R-HRQoL scoring is defined as the difference (Thy-R-HRQoL difference) and estimated by subtracting the pre-treatment results from the post-treatment ones, hence a negative difference was defined as a post-treatment improvement.

To check the reliability of the Thy-R-HRQoL questionnaire we used Cronbach’s Alpha Factor, both initially and after obtaining the results. Our decision to test the reliability was because it was an author’s psychometric test and had never been verified before, in contrast to the SF-36 test (Table 3). Initially, the questionnaire contained 51 questions; however, we removed 2 of them, as their context differed from others in the domain and also reflected in Cronbach’s alpha test.

The differences between mean values were examined with a parametric paired *t* test for dependent samples or Welch’s *t* test for independent samples. The direction and strength of interdependence between the chosen variables were examined with Pearson’s correlations coefficients.

The level of significance was set at α = 0.05.

All statistical analyses were performed using STATISTICA StatSoft, Inc. (2011). STATISTICA (data analysis software system), version 10 (www.statsoft.com).

## Results

The result of NTG radioiodine treatment previously reported by us was mean goiter size reduction of 43.18% (range: −17.23 to 89.66%, median: 47.02%) 1 year after treatment (3). In absolute values, mean thyroid volume before treatment was 104.36 ml (median: 92.04, range: 36.23–301.09), and decreased to 58.66 ml (median: 53.20 ml, range: 10.82–203.32 ml) after treatment, differing significantly from the pre-treatment value (*p* = 0.0001). Mean TSH before 131-I treatment was 0.76, with the range of 0.01–4.11 μIU/ml. In this group, 6 patients (17.1%) required LT4 supplementation. After treatment, mean TSH was 1.48 with the range of 0.03–4.15 μIU/ml, and 15 patients (42.9%) required L-T4 substitution. Mean dose for patients taking l-thyroxine before 131-I treatment was 53.87 μg (for the group administered LT4, the mean was 9.23 μg for the whole group) and 91.31 μg after treatment (for the group administered LT4, the mean was 39.13 μg for the whole group). Four patients required a dose increase after 131-I. No statistically significant SF-36 and Thy-R-HRQoL score differences were observed between l-thyroxine substituting and non-substituting groups, both before and after 131-I treatment.

The results of the SF-36 test right before and 12 months after 131-I treatment are presented in Table 1. We observed an improvement in six out of eight SF-36 subscales: PF, RP, RE, VT, SF, and General Health (GH). Comparing SF-36 answers provided by pre-treatment patients and the control group, we found statistically significant differences only in two domains: PF and VT. Comparison of SF-36 results between post-131-I patients and control subjects revealed a noticeable improvement only for the BP domain (Table 1). We attempted to verify if there was any correlation between SF-36 QoL improvement (SF-36 difference) and the percentage of goiter size reduction. We found a correlation within only one domain, a negative correlation between goiter reduction and BP. Results are presented in Table 2. Pearson’s correlation coefficients for the goiter size (milliliter) and SF-36 scales before the treatment indicated that there was a correlation only for RP [*r* = −0.395 (*p* = 0.019)], whereas no statistically significant correlation was found for the remaining seven out of eight subscales [all parameters before treatment: PF −0.177 (*p* = 0.308), RE-0.145 (*p* = 0.406), VT 0.139 (*p* = 0.426), MH −0.022 (*p* = 0.902), SF −0.145 (*p* = 0.405), BP 0.050 (*p* = 0.775), and GH 0.081 (*p* = 0.642)]. Pearson’s correlation coefficients for the changes in SF-36 (SF-36 differences) scales and goiter size before treatment (milliliter) were all statistically insignificant (Table 2). While investigating whether age of patients had any impact on SF-36 improvement (SF-36 difference), we found a negative correlation between changes in SF-36 and patient’s age in three out of eight scales: PF, VT, and SF. In these scales, the older the patient was, the smaller SF-36 difference was observed (Table 2).

**Table 1:** 36-item Short Form (SF-36) and Thyroid-Related Health-Related Quality of Life (Thy-R-HRQoL) scores results before, after 131-I treatment, and in the control group and statistical significance of changes.

Variable	Mean ± SD			*p*-Value		
		
	Before	After	Control	Before vs After	Before vs Control	After vs Control
**SF-scales**						
Physical Functioning	50.86 ± 25.01	62.86 ± 26.85	62.70 ± 24.71	0.027	0.047	0.980
Role-Physical	39.29 ± 42.57	60.71 ± 42.13	53.38 ± 40.89	0.026	0.157	0.456
Role-Emotional	49.52 ± 44.55	69.52 ± 42.30	61.26 ± 46.16	0.045	0.276	0.431
Vitality	46.86 ± 15.68	56.29 ± 16.82	56.35 ± 16.27	0.006	0.014	0.987
Mental Health	58.17 ± 12.32	63.77 ± 15.25	60.00 ± 16.30	0.097	0.592	0.314
Social Functioning	60.71 ± 23.32	70.71 ± 20.99	69.26 ± 21.57	0.021	0.112	0.772
Bodily Pain	57.71 ± 24.01	66.43 ± 24.49	53.24 ± 23.88	0.124	0.431	0.024
General Health (GH)	40.14 ± 9.81	43.86 ± 9.86	42.30 ± 10.97	0.037	0.382	0.527

**Thy-R-HRQoL**						
Disease and its influence on GH status and social role functioning	3.58 ± 0.98	3.41 ± 0.86	1.85 ± 0.93	0.238	<0.001	<0.001
Neck shape and deformities and its influence on GH status and social role functioning	2.73 ± 0.93	2.54 ± 1.13	1.70 ± 0.73	0.380	<0.001	<0.001
Dyspnea and its influence on GH status and social role functioning	3.66 ± 1.21	2.99 ± 1.19	1.82 ± 0.92	0.003	<0.001	<0.001
Signs of breath impairment	4.15 ± 1.23	3.47 ± 1.17	1.88 ± 1.05	0.002	<0.001	<0.001
Feeling of a foreign body in the throat	3.34 ± 1.33	2.80 ± 1.22	1.78 ± 1.00	0.032	<0.001	<0.001
Difficulty swallowing and hoarseness	3.70 ± 1.51	3.34 ± 1.50	1.97 ± 1.22	0.096	<0.001	<0.001
Other	3.00 ± 0.86	3.07 ± 0.83	1.94 ± 1.08	0.624	<0.001	<0.001

*In SF-36, the higher the score, the greater the satisfaction, and the lower the score, the higher the disability. In Thy-R-HRQoL, the higher the score of the test, the greater the impact of the disease on reported ailments. Results Before—test scores before 131-I treatment in patients with nontoxic goiter (NTG), After—test scores after 131-I treatment in patients with NTG, Control—test scores obtained from the control group*.

**Table 2:** Correlation between percentage goiter size reduction, patients’ age, initial goiter size, and changes in 36-item Short Form and Thyroid-Related Health-Related Quality of Life (Thy-R-HRQoL) scales.

	Goiter size reduction (%)		Patients’ age (years)		Initial goiter size (ml)	
			
Difference in SF-scales	*r*	*p*-Value	*r*	*p*-Value	*r*	*p*-Value
Physical Functioning	−0.023	0.895	−0.421	0.012	0.003	0.986
Role-Physical	−0.014	0.936	−0.128	0.463	0.285	0.097
Role-Emotional	0.025	0.889	−0.230	0.184	0.122	0.486
Vitality	−0.184	0.290	−0.350	0.039	−0.032	0.853
Mental Health	−0.036	0.836	0.010	0.956	−0.085	0.626
Social Functioning	0.016	0.929	−0.500	0.002	−0.080	0.649
Bodily Pain	−0.396	0.019	−0.287	0.095	−0.084	0.631
General Health (GH)	0.008	0.962	−0.120	0.493	0.070	0.689
**Difference in Thy-R-HRQoL**						
Disease and its influence on GH status and social role functioning	−0.153	0.396	0.080	0.657	−0.179	0.318
Neck shape and deformities and its influence on GH status and social role functioning	0.068	0.708	−0.020	0.911	−0.183	0.308
Dyspnea and its influence on GH status and social role functioning	−0.068	0.706	0.010	0.957	−0.032	0.861
Signs of breath impairment	−0.048	0.791	0.051	0.776	−0.084	0.642
Feeling of a foreign body in the throat	−0.064	0.725	0.232	0.193	−0.020	0.912
Difficulty swallowing and hoarseness	−0.152	0.399	0.121	0.503	0.319	0.070
Other	−0.010	0.954	0.140	0.436	−0.111	0.538

*Pearson correlation coefficients (symbol *r*) were significant at *p*<0.05*.

To check the reliability of data collected using Thy-R-HRQoL, we assessed the internal consistency of items (6). Cronbach’s alpha values for all factors were high both before and after the treatment (over 0.9 Table 3). In three out of seven scales of Thy-R-HRQoL, we observed an improvement of QoL. The results of Thy-R-HRQoL before and after 131-I treatment are presented in Table 1. Assessment of Thy-R-HRQoL score differences between NTG patients (before and after 131-I) and the control group resulted in finding a noticeable trend—the NTG group reported lower scores in all domains of Thy-R-HRQoL (Table 1).

**Table 3:** Cronbach’s alpha values for Thyroid-Related Health-Related Quality of Life before and after treatment.

Item	Cronbach’s alpha value	
	
	Before	After
Disease and its influence on general health (GH) status and social role functioning	0.939	0.939
Neck shape and deformities and its influence on GH status and social role functioning	0.917	0.958
Dyspnea and its influence on GH status and social role functioning	0.936	0.942
Signs of breath impairment	0.913	0.928
Feeling of a foreign body in the throat	0.763	0.864
Difficulty swallowing and hoarseness	0.900	0.981
Other	0.782	0.791
All	0.960	0.956

We did not observe any correlation between changes in Thy-R-HRQoL (Thy-R-HRQoL difference) and results of the treatment represented by goiter size reduction (Table 2). Pearson’s correlation coefficients for goiter size (milliliter) and Thy-R-HRQoL scores (both before treatment) indicated that there was a correlation for “neck shape and deformities and its influence on GH status and social role functioning” only. The greater the goiter size before treatment, the higher (worse) scores for neck shape and deformities and the more observable their influence on GH status and social role functioning [*r* = 0.528 (*p* = 0.002)]. For the remaining six out of seven scales, we did not notice any statistically significant correlations [disease and its influence on GH status and social role functioning *r* = 0.174 (*p* = 0.334), dyspnea and its influence on GH status and social role functioning *r* = 0.033 (*p* = 0.854), signs of breathing impairment *r* = 0.081 (*p* = 0.653), feeling of a foreign body in the throat *r* = 0.130 (*p* = 0.471), difficulty swallowing and hoarseness *r* = −0.024 (*p* = 0.896), other *r* = 0.188 (*p* = 0.296)]. Pearson’s correlation coefficients for changes in Thy-R-HRQoL (Thy-R-HRQoL difference) and goiter size before treatment (milliliter) were all statistically insignificant (Table 2). Correlations between patient age and changes in Thy-R-HRQoL scores (Thy-R-HRQoL difference) were statistically insignificant for all subscales (Table 2).

Mean age among NTG patients and the control group did not differ significantly (*p* = 0.215).

## Discussion

We attempted to verify whether the arguments for administration of 131-I in NTG are sufficient to be reflected in patient’s satisfaction with the treatment. Our research into QoL in patients with NTG prior to and after radioiodine treatment resulted in the conclusion that radioiodine treatment improves their life quality. We believe that the evaluation of life quality using general QoL tests may not be sufficient and therefore HRQoL tests in patients with NTG should be used.

Promberger et al. reported in their SF-36 assessment that after thyroid surgery in women with NTG, the SF-36 increased only in the BP domain (7). In the group undergoing thyroid surgery, Miccoli et al. observed improvement only in the MH of SF-36. In comparison to the results before thyroid surgery from the normative Italian sample, they found impairment in SF and significant decrease in the RP domain (8). Cramon et al. did not observe improvement in any of the SF-36 domains: patients treated with radioiodine (32%), hemithyroidectomy (53%), total thyroidectomy (12%), or cyst aspiration with ethanol sclerotherapy (4%). Although at baseline, in four out of eight SF-36 scales patients had worse scores than the general population (9). We observed an improvement in most (six out of eight) SF-36 scales, the only exceptions being MH and BP. However, comparing SF-36 results of patients before 131-I treatment to the control group, we initially observed a decrease only in PF and VT, which was not observed by the patients after the treatment. BP was the only parameter which was different in our group of patients after treatment from the control group. The results may suggest a subjective sense of improvement in patients with NTG following treatment with QoL slightly decreased in comparison with the general population.

SF-36 is a tool used to evaluate BP, general sense of well-being and everyday functioning in society. The results of our study, similar to those obtained by the authors cited above, throw into question whether SF-36 itself is sufficient for the evaluation of QoL in NTG (7, 9). They suggest the need for an evaluation through HRQoL. We attempted to develop such a scale by creating the Thy-R-HRQoL. At the beginning of our study, we did not have access to any other questionnaires. Unfortunately, the Thyroid Patient-Reported Outcome (Thy-PRO), which is currently proposed by the Danish researchers, was still in the making at that time (10, 11).

While creating the test we focused on the evaluation of specific domains in terms of thyroid enlargement. We observed a deterioration of QoL in all Thy-R-HRQoL domains, both prior to treatment and afterward in comparison with the control group. Such results suggest that NTG impairs QoL in comparison with the healthy population. They also suggest that the HRQoL test is necessary to focus on the specific symptoms of NTG and is a more suitable tool to assess patients’ life quality than general QoL evaluation tests.

It appears that the greatest benefits of 131-I treatment were related to the resolution of compressive symptoms. After treatment, we observed an improvement in three out of seven scales: dyspnea, signs of breath impairment, and feeling of a foreign body in the throat. Lack of improvement in other domains does not necessarily prove the therapy to be unsuccessful. It should be taken into consideration that after 131-I treatment the disease does not disappear. The goiter size decreases yet is still present. Not all the symptoms resolve and if they do, they may not be resolved completely. It must be emphasized that even in the domains with a reported post-treatment improvement our patients obtained different (worse) results than the control group. We consider this outcome as an indirect proof of the specificity of our test.

At the same time, while we were using our own Thy-R-HRQoL, Danish researchers developed the thyroid-specific patient-reported outcome (ThyPRO) (12). In the ThyPRO assessment of benign thyroid goiters in patients after total thyroidectomy (54%) or hemithyroidectomy (46%), an improvement was observed in 12 out of 13 scales (13). In a group of patients with benign NTG treated with radioiodine (32%) or surgery (65%), Cramon et al. reported an improvement in 6 out of 13 ThyPRO scales (9). In 9 out of 13 baseline scores and in 8 out of 13 post-treatment scores, their results were significantly worse than those of the general population (9).

We were unable to prove that patients’ satisfaction with the treatment depends directly on the degree of thyroid reduction. Whether goiter volume reduction is in fact the most significant factor affecting QoL in patients with NTG remains questionable. We checked the correlation between the degree of goiter reduction and the improvement in certain domains in SF-36 and Thy-R-HRQoL. Except for the BP change, there was no correlation with the extension of goiter size reduction and SF-36 improvement. Additionally, no correlation between test results improvement and goiter reduction was found in our Thy-R-HRQoL assessment. We might suggest that a patient with an insignificant reduction and a patient with a 90% reduction can be equally satisfied with treatment results. This should be taken into consideration while evaluating 131-I treatment results of NTG patients as even an insignificant goiter reduction may considerably facilitate QoL improvement. Other factors, apart from goiter reduction, such as further thyroid enlargement prevention, sense of control or being cared for, and having an alternative to surgery, may all positively affect post-131-I treatment QoL improvement in patients with NTG.

We also posed a question of whether there are any factors which interfere with the evaluation of the treatment afterward.

We attempted to answer the question of, whether patients with an initially larger goiter reported a greater post-treatment QoL improvement. Could initially greater thyroid volume and possibly more intense symptoms be the reason for greater satisfaction with the treatment? There was no correlation between the Thy-R-HRQoL and SF-36 improvement and the initial goiter size.

We also wanted to find out whether patients’ age may affect their expectations regarding treatment results. It may appear that expectations regarding an improvement in the neck shape of either a decrease in exertional or at rest dyspnea due to different activities may be different in different age groups. In our study, the older the patient was, the lesser the extent of improvement for PF, VT, and SF scales was observed. PF and VT were the only domains which initially differed significantly in the NTG group in comparison with the general population. It is difficult to conclusively determine whether a worse evaluation of the treatment effects results because of age itself or from the initially worse evaluation of the parameters. It is hard to say if it can be directly correlated with NTG and 131-I treatment. In the evaluation of the symptoms specific for NTG (Thy-R-HRQoL), we did not observe any correlation between reported post-treatment improvement and the age of the patients.

The last question we posed, which was not related to the evaluation of treatment results was whether patients with initially larger goiter size complained of initially lower QoL. Our study suggests that only regarding RP, the greater the initial size, the lower SF-36 assessment score was obtained. With the Thy-R-HRQoL assessment, we found that the larger the goiter, the greater influence of neck shape and deformities on GH status and social role functioning. However, no clear impact on dyspnea, signs of breathing impairment, or any of the remaining scales was documented. We may speculate that the size of the goiter is not the main factor deteriorating QoL in patients with NTG (which seems to be consistent in some way, with the above conclusions that patients’ satisfaction with the treatment does not depend on the degree of thyroid reduction).

We also tried to find whether hypothyroidism before and after treatment affected therapeutic outcomes. l-thyroxine substituting and non-substituting patients presented statistically similar SF-36 and Thy-R-HRQoL test results both before and after treatment.

The study was significantly limited by the small size of the research group but then homogeneic in terms of treatment methods. Our small, carefully selected group of patients makes drawing definite conclusions risky. It is difficult to draw conclusions if there are no other publications available for comparison. It is difficult to find publications for comparison if there are no HRQoL tests available. We regret that ThyPRO was unavailable to us at the beginning of our research. At the same time, with an ever-increasing interest in the subject of QoL in patients with NTG, we truly hope that a greater number of tests, including our Thy-R-HRQoL, will prove helpful in future research.

## Conclusion

As far as we can judge, despite our study’s limitations, we would like to conclude that radioiodine treatment improves general life quality in patients with NTG. Tests available for the general QoL evaluation may be helpful, although in the case of patients with NTG, HRQoL tests are indispensable. We would like to turn this study into an opportunity to start a discussion aimed at answering the question whether relentless pursuit of maximal goiter size reduction *via* 131-I treatment is necessary and whether the initial goiter size is the major component of QoL evaluation in patients with NTG. Life quality improvement probably does not depend on the goiter reduction and initial goiter size. It seems that only in the assessment of the general QoL (PF, VT, and SF scales), the test results may vary depending on the patient’s age.

The results we obtained certainly cannot be looked upon as definite proof, however, in light of limited literary reports may be seen as a voice to add to the discussion of this topic.

## Ethics Statement

The scheme of the study and treatment gained consent from the MUG Independent Bioethics Commission.

## Author Contributions

SK-J drafted the manuscript. SK-J, AL, MM, MP, KM-G and AZ were involved in collecting the group, treatment, performed the tests, and gathered the tests results. SK-J and MK were involved in statistical analysis, PL and KS supervised the study and reviewed the manuscript.

## Conflict of Interest Statement

The authors declare that the research was conducted in the absence of any commercial or financial relationships that could be construed as a potential conflict of interest.
