# Supplementary material for: Nontoxic Goiter (NTG) and Radioiodine: What Do Patients Think About It? Quality of Life in Patients with NTG Before and After 131-I Therapy
Source: Front Endocrinol (Lausanne). 2018 Apr 16;9:114. doi: 10.3389/fendo.2018.00114 (PMC5912045; doi:10.3389/fendo.2018.00114)
Supplement: Supplementary file 1 [file data_sheet_1.DOC]

Welcome!

We would like to find out **how the thyroid disease affects your general well-being** and therefore we would like to ask you to answer a few questions.

- Read every question carefully
- Think of how the disease affects you, what your situation looked like prior to the onset of the disease and what it currently looks like
- Choose the answer which best suits you and mark it with X

Remember that there are no bad or good answers in this survey. We would just appreciate if you wrote what you think.

**EXAMPLE:** Or how to do the survey.

Do you feel that the thyroid disease affects your everyday well-being?

If you think that the thyroid disease **definitely affects** your well-being, put a cross under the last answer on the right.

| **DEFINITELY NO**   |  | | --- | | **NO**   |  | | --- | | **RATHER NO**   |  | | --- | | **RATHER YES**   |  | | --- | | **YES**   |  | | --- | | **DEFINITELY YES**   |  | | --- | |
| --- | --- | --- | --- | --- | --- | --- | --- | --- | --- | --- | --- |

If you think that the thyroid disease **definitely does not affect** your well-being, put a cross under the first answer on the left.

| **DEFINITELY NO**   |  | | --- | | **NO**   |  | | --- | | **RATHER NO**   |  | | --- | | **RATHER YES**   |  | | --- | | **YES**   |  | | --- | | **DEFINITELY YES**   |  | | --- | |
| --- | --- | --- | --- | --- | --- | --- | --- | --- | --- | --- | --- |

If you think that the thyroid disease **rather affects** your well-being, in the fourth square.

| **DEFINITELY NO**   |  | | --- | | **NO**   |  | | --- | | **RATHER NO**   |  | | --- | | **RATHER YES**   |  | | --- | | **YES**   |  | | --- | | **DEFINITELY YES**   |  | | --- | |
| --- | --- | --- | --- | --- | --- | --- | --- | --- | --- | --- | --- |

If you think that the thyroid disease **does not rather affect** your well-being, put a cross in the third square.

| **DEFINITELY NO**   |  | | --- | | **NO**   |  | | --- | | **RATHER NO**   |  | | --- | | **RATHER YES**   |  | | --- | | **YES**   |  | | --- | | **DEFINITELY YES**   |  | | --- | |
| --- | --- | --- | --- | --- | --- | --- | --- | --- | --- | --- | --- |

If you think that the thyroid disease **DOES NOT affect** your well-being, put a cross in the second square.

| **DEFINITELY NO**   |  | | --- | | **NO**   |  | | --- | | **RATHER NO**   |  | | --- | | **RATHER YES**   |  | | --- | | **YES**   |  | | --- | | **DEFINITELY YES**   |  | | --- | |
| --- | --- | --- | --- | --- | --- | --- | --- | --- | --- | --- | --- |

If you think that the thyroid disease **affects** your well-being, put a cross in the fifth square.

| **DEFINITELY NO**   |  | | --- | | **NO**   |  | | --- | | **RATHER NO**   |  | | --- | | | **RATHER YES**   |  | | --- | | | | **YES**   |  | | --- | | | | **DEFINITELY YES**   |  | | --- | | | |
| --- | --- | --- | --- | --- | --- | --- | --- | --- | --- | --- | --- | --- | --- | --- | --- | --- | --- | --- |
|  | | | **DEFINITELY**  **NO** | | **NO** | **RATHER NO** | | **RATHER YES** | **YES** | | **DEFINITELY**  **YES** |  |
| **DISEASE AND ITS INFLUENCE ON GENERAL HEALTH STATUS AND SOCIAL ROLE FUNCTIONING** | | | | | | | | | | | |  |
| Does your thyroid disease affect your general sense of health? | | | |  | | --- | | | |  | | --- | | |  | | --- | | | |  | | --- | | |  | | --- | | | |  | | --- | |  |
| Has your thyroid disease affected your general well-being? | | | |  | | --- | | | |  | | --- | | |  | | --- | | | |  | | --- | | |  | | --- | | | |  | | --- | |  |
| Has your thyroid disease changed your life in any way? | | | |  | | --- | | | |  | | --- | | |  | | --- | | | |  | | --- | | |  | | --- | | | |  | | --- | |  |
| Has your thyroid disease affected your life’s focus | | | |  | | --- | | | |  | | --- | | |  | | --- | | | |  | | --- | | |  | | --- | | | |  | | --- | |  |
| Has your thyroid disease affected your general functioning? | | | |  | | --- | | | |  | | --- | | |  | | --- | | | |  | | --- | | |  | | --- | | | |  | | --- | |  |
| Has your thyroid disease affected your work? | | | |  | | --- | | | |  | | --- | | |  | | --- | | | |  | | --- | | |  | | --- | | | |  | | --- | |  |
| Has your thyroid disease affected your functioning in the family? | | | |  | | --- | | | |  | | --- | | |  | | --- | | | |  | | --- | | |  | | --- | | | |  | | --- | |  |
| Has your thyroid disease affected your functioning in the society? | | | |  | | --- | | | |  | | --- | | |  | | --- | | | |  | | --- | | |  | | --- | | | |  | | --- | |  |
| Has your thyroid disease lowered your self-assessment? | | | |  | | --- | | | |  | | --- | | |  | | --- | | | |  | | --- | | |  | | --- | | | |  | | --- | |  |
| Has your thyroid disease lowered your self-esteem? | | | |  | | --- | | | |  | | --- | | |  | | --- | | | |  | | --- | | |  | | --- | | | |  | | --- | |  |
| May your thyroid disease trigger or increase your anxiety? | | | |  | | --- | | | |  | | --- | | |  | | --- | | | |  | | --- | | |  | | --- | | | |  | | --- | |  |
| May your thyroid disease increase your edginess? | | | |  | | --- | | | |  | | --- | | |  | | --- | | | |  | | --- | | |  | | --- | | | |  | | --- | |  |
| Has the thyroid disease increased your sense of tiredness? | | | |  | | --- | | | |  | | --- | | |  | | --- | | | |  | | --- | | |  | | --- | | | |  | | --- | |  |
| Does the thyroid disease cause sexual problems? | | | |  | | --- | | | |  | | --- | | |  | | --- | | | |  | | --- | | |  | | --- | | | |  | | --- | |  |
| Does your thyroid disease decreases your self-confidence? | | | |  | | --- | | | |  | | --- | | |  | | --- | | | |  | | --- | | |  | | --- | | | |  | | --- | |  |
| May your thyroid disease increase your tiredness and sleepiness? | | | |  | | --- | | | |  | | --- | | |  | | --- | | | |  | | --- | | |  | | --- | | | |  | | --- | |  |
| **NECK SHAPE AND DEFORMITIES AND ITS INFLUENCE ON GENERAL HEALTH STATUS AND SOCIAL ROLE FUNCTIONING** | | | | | | | | | | | |  |
|  | | | **DEFINITELY NO** | | **NO** | **RATHER NO** | | **RATHER YES** | **YES** | | **DEFINITELY YES** |  |
| Has neck deformation affected your everyday life’s focus? | | | |  | | --- | | | |  | | --- | | |  | | --- | | | |  | | --- | | |  | | --- | | | |  | | --- | |  |
| Has neck deformation affected your self-assessment? | | | |  | | --- | | | |  | | --- | | |  | | --- | | | |  | | --- | | |  | | --- | | | |  | | --- | |  |
| Has neck deformation affected your general sense of health? | | | |  | | --- | | | |  | | --- | | |  | | --- | | | |  | | --- | | |  | | --- | | | |  | | --- | |  |
| Has neck deformation affected your interactions with other people? | | | |  | | --- | | | |  | | --- | | |  | | --- | | | |  | | --- | | |  | | --- | | | |  | | --- | |  |
|  | | |  | |  |  | |  |  | |  |  |
| Has neck deformation affected your work? | | | |  | | --- | | | |  | | --- | | |  | | --- | | | |  | | --- | | |  | | --- | | | |  | | --- | |  |
| Has neck deformation affected your functioning in the family? | | | |  | | --- | | | |  | | --- | | |  | | --- | | | |  | | --- | | |  | | --- | | | |  | | --- | |  |
| Has neck deformation affected your self-confidence? | | | |  | | --- | | | |  | | --- | | |  | | --- | | | |  | | --- | | |  | | --- | | | |  | | --- | |  |
| Has neck deformation affected your self-esteem? | | | |  | | --- | | | |  | | --- | | |  | | --- | | | |  | | --- | | |  | | --- | | | |  | | --- | |  |
| Do you think that your neck deformation is noticeable to other people? | | | |  | | --- | | | |  | | --- | | |  | | --- | | | |  | | --- | | |  | | --- | | | |  | | --- | |  |
| Does it bother you that others notice a change in your neck shape? | | | |  | | --- | | | |  | | --- | | |  | | --- | | | |  | | --- | | |  | | --- | | | |  | | --- | |  |
| Do you think that neck deformation has affected your general physical appearance? | | | |  | | --- | | | |  | | --- | | |  | | --- | | | |  | | --- | | |  | | --- | | | |  | | --- | |  |
| Do you sometimes have the impression that others look at your neck? | | | |  | | --- | | | |  | | --- | | |  | | --- | | | |  | | --- | | |  | | --- | | | |  | | --- | |  |
| **DYSPNOEA AND ITS INFLUENCE ON GENERAL HEALTH STATUS AND SOCIAL ROLE FUNCTIONING** | | | | | | | | | | | |  |
|  | | | **DEFINITELY NO** | | **NO** | **RATHER NO** | | **RATHER YES** | **YES** | | **DEFINITELY YES** |  |
| Does your thyroid disorder cause breathing disorders? | | | |  | | --- | | | |  | | --- | | |  | | --- | | | |  | | --- | | |  | | --- | | | |  | | --- | |  |
| Do breathing disorders affect your everyday life’s focus? | | | |  | | --- | | | |  | | --- | | |  | | --- | | | |  | | --- | | |  | | --- | | | |  | | --- | |  |
| Do breathing disorders affect your general sense of health? | | | |  | | --- | | | |  | | --- | | |  | | --- | | | |  | | --- | | |  | | --- | | | |  | | --- | |  |
| Do breathing disorders affect your interactions with other people? | | | |  | | --- | | | |  | | --- | | |  | | --- | | | |  | | --- | | |  | | --- | | | |  | | --- | |  |
| Do breathing disorders affect your work? | | | |  | | --- | | | |  | | --- | | |  | | --- | | | |  | | --- | | |  | | --- | | | |  | | --- | |  |
| Do breathing disorders affect your functioning in the family? | | | |  | | --- | | | |  | | --- | | |  | | --- | | | |  | | --- | | |  | | --- | | | |  | | --- | |  |
| Do breathing disorders increase your tiredness during the day? | | | |  | | --- | | | |  | | --- | | |  | | --- | | | |  | | --- | | |  | | --- | | | |  | | --- | |  |
| **SIGNS OF BREATH IMPAIRMENT** | | | | | | | | | | | |  |
|  | | | **DEFINITELY NO** | | **NO** | **RATHER NO** | | **RATHER YES** | **YES** | | **DEFINITELY YES** |  |
| Does your thyroid disease cause accelerated respiration of shortness/ shallowness of breath? | | | |  | | --- | | | |  | | --- | | |  | | --- | | | |  | | --- | | |  | | --- | | | |  | | --- | |  |
| Does your thyroid disease cause sense of breathlessness? | | | |  | | --- | | | |  | | --- | | |  | | --- | | | |  | | --- | | |  | | --- | | | |  | | --- | |  |
| Does your thyroid disease cause shortness of breath more frequently? | | | |  | | --- | | | |  | | --- | | |  | | --- | | | |  | | --- | | |  | | --- | | | |  | | --- | |  |
|  | | |  | |  |  | |  |  | |  |  |
| Does your thyroid disease make you feel tired more quickly and easily? | | | |  | | --- | | | |  | | --- | | |  | | --- | | | |  | | --- | | |  | | --- | | | |  | | --- | |  |
| **FEELING OF A FOREIGN BODY IN THE THROAT** | | | | | | | | | | | |  |
|  | | | **DEFINITELY NO** | | **NO** | **RATHER NO** | | **RATHER YES** | **YES** | | **DEFINITELY YES** |  |
| Does your thyroid disease cause a sense of foreign body in your throat? | | | |  | | --- | | | |  | | --- | | |  | | --- | | | |  | | --- | | |  | | --- | | | |  | | --- | |  |
| **DIFFICULTY SWALLOWING, AND HOARSENESS** | | | | | | | | | | | |  |
|  | | | **DEFINITELY NO** | | **NO** | **RATHER NO** | | **RATHER YES** | **YES** | | **DEFINITELY YES** |  |
| Does your thyroid disease cause swallowing difficulties? | | | |  | | --- | | | |  | | --- | | |  | | --- | | | |  | | --- | | |  | | --- | | | |  | | --- | |  |
| Has your thyroid disease caused a change in your vocal tone? | | | |  | | --- | | | |  | | --- | | |  | | --- | | | |  | | --- | | |  | | --- | | | |  | | --- | |  |
| Has your thyroid disease caused or increased hoarseness? | | | |  | | --- | | | |  | | --- | | |  | | --- | | | |  | | --- | | |  | | --- | | | |  | | --- | |  |
| **OTHER** | | | | | | | | | | | |  |
| Has your thyroid disease affected your weight? | | | |  | | --- | | | |  | | --- | | |  | | --- | | | |  | | --- | | |  | | --- | | | |  | | --- | |  |
| Has your thyroid disease caused muscular system disorders? | | | |  | | --- | | | |  | | --- | | |  | | --- | | | |  | | --- | | |  | | --- | | | |  | | --- | |  |
| Has your thyroid disease caused disorders of the skeleto-muscular system? | | | |  | | --- | | | |  | | --- | | |  | | --- | | | |  | | --- | | |  | | --- | | | |  | | --- | |  |
| Does your thyroid disease cause any pain? | | | |  | | --- | | | |  | | --- | | |  | | --- | | | |  | | --- | | |  | | --- | | | |  | | --- | |  |
| Has your thyroid disease caused bowel disorders? | | | |  | | --- | | | |  | | --- | | |  | | --- | | | |  | | --- | | |  | | --- | | | |  | | --- | |  |
| Has your thyroid disease caused or increased any heart conditions? | | | |  | | --- | | | |  | | --- | | |  | | --- | | | |  | | --- | | |  | | --- | | | |  | | --- | |  |
